# Proteomic Identification of Plasma Protein Tyrosine Phosphatase Alpha and Fibronectin Associated with Liver Fluke, *Opisthorchis viverrini*, Infection

**DOI:** 10.1371/journal.pone.0045460

**Published:** 2012-09-18

**Authors:** Jarinya Khoontawad, Umawadee Laothong, Sittiruk Roytrakul, Porntip Pinlaor, Jason Mulvenna, Chaisiri Wongkham, Puangrat Yongvanit, Chawalit Pairojkul, Eimorn Mairiang, Paiboon Sithithaworn, Somchai Pinlaor

**Affiliations:** 1 Department of Parasitology, Faculty of Medicine, Khon Kaen University, Khon Kaen, Thailand; 2 Proteomics Research Laboratory, Genome Institute Biotechnology, Pathumthani, Thailand; 3 Centre for Research and Development in Medical Diagnostic Laboratory, Faculty of Associated Medical Sciences, Khon Kaen University, Khon Kaen, Thailand; 4 Department of Biochemistry, Faculty of Medicine, Khon Kaen University, Khon Kaen, Thailand; 5 Department of Pathology, Faculty of Medicine, Khon Kaen University, Khon Kaen, Thailand; 6 Department of Radiology, Faculty of Medicine, Khon Kaen University, Khon Kaen, Thailand; 7 Liver Fluke and Cholangiocarcinoma Research Center, Faculty of Medicine, Khon Kaen University, Khon Kaen, Thailand; 8 Department of Infectious Disease and Cancer, Faculty of Computational Biology, Queensland Institute of Medical Research, Brisbane, Queensland, Australia; Centro de Investigacion y de Estudios Avanzados del Instituto Politecnico Nacional, Mexico

## Abstract

Opisthorchiasis caused by *Opisthorchis viverrini* induces periductal fibrosis via host immune/inflammatory responses. Plasma protein alteration during host-parasite interaction-mediated inflammation may provide potential diagnostic and/or prognostic biomarkers. To search for target protein changes in *O. viverrini*-infected hamsters, a 1-D PAGE gel band was trypsin-digested and analyzed by a LC-MS/MS-based proteomics approach in the plasma profile of infected hamsters, and applied to humans. Sixty seven proteins were selected for further analysis based on at least two unique tryptic peptides with protein ID score >10 and increased expression at least two times across time points. These proteins have not been previously identified in *O. viverrini*-associated infection. Among those, proteins involved in structural (19%), immune response (13%), cell cycle (10%) and transcription (10%) were highly expressed. Western blots revealed an expression level of protein tyrosine phosphatase alpha (PTPα) which reached a peak at 1 month and subsequently tended to decrease. Fibronectin significantly increased at 1 month and tended to increase with time, supporting proteomic analysis. PTPα was expressed in the cytoplasm of inflammatory cells, while fibronectin was observed mainly in the cytoplasm of fibroblasts and the extracellular matrix at periductal fibrosis areas. In addition, these protein levels significantly increased in the plasma of *O. viverrini*-infected patients compared to healthy individuals, and significantly decreased at 2-months post-treatment, indicating their potential as disease markers. **In conclusion**, our results suggest that plasma PTPα and fibronectin may be associated with opisthorchiasis and the hamster model provides the basis for development of novel diagnostic markers in the future.

## Introduction

Opisthorchiasis caused by infection with the liver fluke *Opisthorchis viverrini* remains a major health problem in large parts of Southeast Asia, including Thailand, Lao PDR, Vietnam and Cambodia. Currently, more than 600 million people are estimated to be at risk of infection [1]. Chronic infection with *O. viverrini* is a risk factor for cholangiocarcinoma (CCA), cancer of the bile duct, which is thought to develop as a consequence of chronic inflammation [2], [3]. There is no stronger association between an eukaryotic pathogen and cancer than that between *O. viverrini* and CCA in northeast Thailand, where infection is endemic, and rates of CCA are unprecedented [4].

In acute *O. viverrini* infections the major histopathological changes include the accumulation of inflammatory cells, nitric oxide (NO)-mediated DNA damage and liver tissue injury [5]. This makes diagnosis of *O. viverrini* infection difficult as these changes are clinically silent and generally only detected after ultrasound imaging in severe cases of chronic infection [6]. Thickening of periductal fibrosis is the most prominent histopathological finding after long-term infection and is found in both opisthorchiasis patients [7] and *O. viverrini*-infected hamsters [8]. Moreover, periductal fibrosis increases in hamsters with CCA induced by a combination of *O. viverrini* infection and the administration of a carcinogen (nitrosamine) [9]. Thus, the relationship between periductal fibrosis and CCA suggests that expression changes in biomolecules involved in inflammation, such as those provoked by DNA damage [10], etheno DNA adducts [11] and other fibrotic markers could be used as prognostic marker of opisthorchiasis-associated CCA in humans. Moreover, plasma contains all tissue proteins leakage [12], which may serve as protein-disease markers discovery.

In this study, a proteomics technique was used to search for all possible target protein-disease associations in a hamster model and these were then applied to human subjects. To find target plasma protein changes in *O. viverrini*-infected hamsters, plasma protein was separated using 1-D PAGE, and identified using LC-MS/MS after in-gel tryptic digestion. MS analysis revealed significant changes in the structural protein, fibronectin, and the signal transduction protein, protein tyrosine phosphatase (PTP). These changes were confirmed by western blotting and immunohistochemistry. These two candidate markers were also found to be increased in the plasma of human opisthorchiasis patients and to be decreased after praziquantel treatment. Therefore, plasma proteome of the hamster model is able to provide potential diagnostic biomarkers for opisthorchiasis in humans that could be developed as diagnostic tools for the early detection of opisthorchiasis and, consequently, can act as relative risk factors for *O. viverrini*-induced CCA.

## Materials and Methods

### Parasites

*Opisthorchis viverrini* metacercariae were isolated from naturally infected cyprinid fish by pepsin digestion as described previously [13]. Recently caught and dead cyprinid fish, chilled on ice, were obtained from markets in Khon Kaen Province, Thailand. The fish were digested in 0.25% pepsin-HCl and *O. viverrini* metacercariae were isolated and counted. Viable cysts were used for hamster infection.

### Animals and experimental design

The Animal Ethics Committee of Khon Kaen University approved this study under permit number AEKKU 17/2552. Experiments in an animal model used in this study were conducted and performed in strict accordance with the recommendations in the Guide for the Care and Use of Laboratory Animals of the National Research Council of Thailand. All experimental protocols were approved by the Institutional Animal Ethics Committee, Khon Kaen University and the National Research Council of Thailand for use of laboratory animals. All surgery and necropsy was performed under ether anesthesia, and every effort was made to minimize pain and suffering to the animals.

Male syrian golden hamsters (*Mesocricetus auratus*) (obtained from the Animal Unit, Faculty of Medicine, Khon Kaen University, Khon Kaen, Thailand) aged between 4–6 weeks were used in this study. Hamsters were housed under conventional conditions, fed a stock murine diet (CP-SWT, Thailand), and were given water *ad libitum*. Seventy animals were divided into infected and un-infected groups. In the infected group, animals were infected with 50 metacercariae of *O. viverrini* by intragastric inoculation and were sacrificed at day 21 and 1, 2, 3, 4, 5, and 6 month(s) post-infection, and five animals were included in each subgroups. A normal animal underwent the same procedure as the infected group, minus the metacercariae, and was used as a control.

### Specimen collection

Animals were anaesthetized with ether and blood was collected from the heart. To improve resolution, sensitivity and reproducibility of peptide identifications, we used platelet-depleted plasma samples. For this purpose, ethylenediamine tetra-acetate (EDTA)-blood samples were kept in an ice-box for 2–3 h before centrifugation. Next, they were centrifuged at 3,000×g for 10 min at 4°C and the plasma was stored at −80°C until analysis. Hemolysed samples were excluded in this study [14]–[16]. The hilar region and adjacent areas of the liver were dissected and tissues were placed in 10% buffered formalin. After fixation overnight, they were processed in a conventional manner. Tissue sections (5- µm thickness) were stained with hematoxylin and eosin to evaluate histopathologic changes and used for immunohistochemical study.

### Patient and study design

Written informed consent was obtained from 57 volunteers including healthy individuals (n = 18), and patients with *O. viverrini* infection (n = 39, matched pre- and post-treatment). Subjects in each group were selected on the basis of age- and sex-matched control. *O. viverrini*-infected patients from endemic areas in Khon Kaen Province, Thailand, were examined for parasite eggs in stools by formalin ethyl acetate concentration technique [17]. Periductal fibrosis in opisthorchiasis patient was graded by ultrasonography. The grading score was defined as follows: grade 0 when no echoes were observed; 1+ when echoes were observed in one segment of the liver; and 2+ when echoes were observed in two or three segments of the liver as described previously [7]. Plasma of opisthorchiasis subjects was collected either before or at 2 months post praziquantel treatment. Healthy individuals were confirmed as such by the absence of *O. viverrini* eggs in stool, normal urinary analysis, and normal hepatobiliary tract as evaluated by ultrasonography. Acute infected patients showing positive for nitrate/nitrite and leukocyte in urine were omitted; patients with chronic inflammatory conditions due to hepatitis B virus and TB infections and those with diabetes mellitus were excluded. In addition, among the control candidates, one who had a history of those diseases before 6 months of investigation was excluded. The hemolysed peripheral blood samples were also excluded for measurements. The study protocol was approved by the Khon Kaen University Ethics Committee for Human Research (HE531174).

Peripheral 10 ml blood samples were obtained by sterilized venipuncture, collected in tubes containing heparin and centrifuged at 3000×g for 15 min at 4°C. Plasma was then isolated and stored at −80°C until analysis.

### Workflow of the study

**Figure 1:**
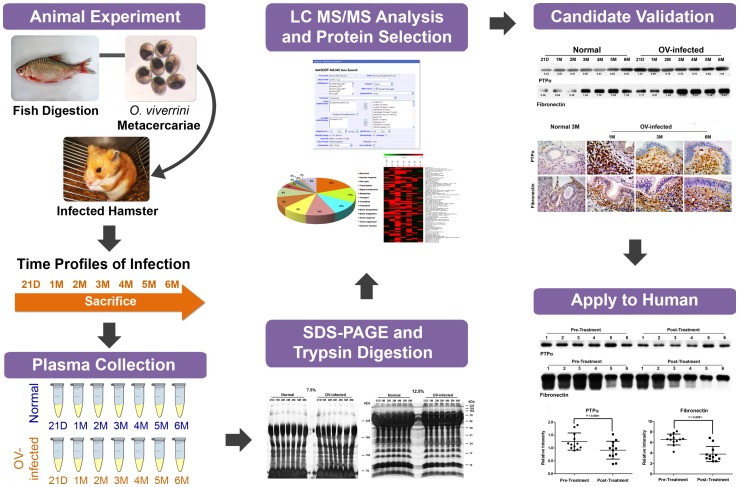
Experimental workflow for the discovery of *Opisthorchis viverrini*-infected plasma biomarkers. After fish digestion, *O. viverrini* metacercariae were collected and fed to hamsters. Animals were sacrificed on day 21, and monthly for 6 months. Pooled plasma samples obtained from five hamsters in each time point were fractionated by SDS-PAGE. Then, entire lanes were excised into slices, digested by trypsin, and were analyzed by LC-MS/MS. After database searching and data analysis, candidate proteins were further validated by western blot and immunohistochemistry. In addition, to apply into human, expression levels of these two candidate markers were also evaluated in the plasma by western blot analysis.

**Figure 2:**
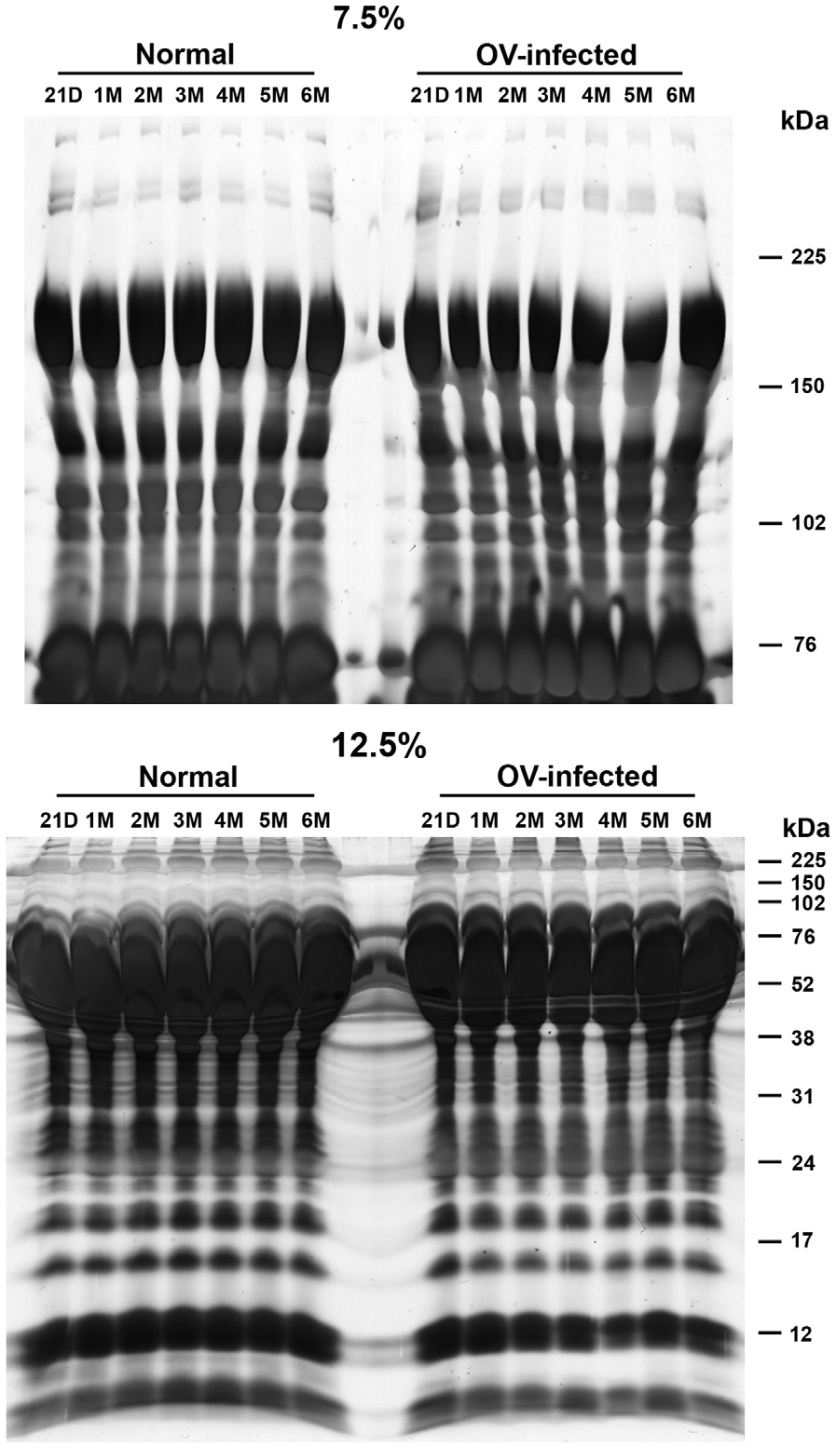
SDS-PAGE fractionation of pooled plasma samples from normal and *Opisthorchis viverrini*-infected groups. Fifty micrograms of pooled plasma protein obtained from 5 animals of each experimental time point was fractionated on a 7.5% and 12.5% acrylamide gel (8×13 cm). Pooled plasma was run on the gel and subsequently stained with silver staining. The each gel lane was sliced and then subjected to in-gel digestion prior to LC/MS analysis. For consistency of results, the assay was done as three independent experiments. Molecular weights of protein marker bands are indicated in kDa on the right of the images. D, days; M, month(s); OV, *Opisthorchis viverrini.*

Figure 1 shows the workflow used to search for alterations in protein expression associated with *O. viverrini*-infected hamsters, and to validate these proteins as potential diagnostic and prognostic biomarkers. Briefly, normal and *O. viverrini*-infected hamster plasma from different time points was fractionated using SDS-PAGE. Next, the gel was silver stained (Figure 2). Gel bands above (in 7.5% gel) and below (in 12.5% gel) albumin band (approximately 76 kDa) were cut, and the resulting proteins were used in LC-MS/MS analysis after in-gel tryptic digestion. Candidate protein markers were validated in hamster plasma and liver sections by western blot and immunohistochemistry. To assess the utility of these animal markers in human subjects, these markers were validated in opisthorchiasis patients using western blot analysis.

### Electrophoresis and tryptic digestion

Plasma protein concentration was measured by Lowrys' method [18]. An equal amount of plasma protein from 5 animals at each time-point of the infected and uninfected groups was pooled. Infected and uninfected plasma protein samples (50 µg each) from each time point were fractionated on both 7.5% and 12.5% polyacrylamide gels (8×13 cm). After silver staining, the bands above albumin in 7.5% gel and below albumin band in 12.5% gel were sliced into 15–18 pieces per lane. Each gel slice was cut into 1 mm pieces, and depending on the size of each slice, approximately 70–90 pieces were digested by trypsin. The gel pieces were subjected to in-gel digestion using an in-house method developed by Proteomics Laboratory, Genome Institute, National Center for Genetic Engineering and Biotechnology (BIOTEC), National Science and Technology Development Agency (NSTDA), Thailand. Briefly, the gel slices were dehydrated with 100% acetonitrile (ACN), reduced with 10 mM DTT in 10 mM ammonium bicarbonate at room temperature for 1 h and alkylated at room temperature for 1 h in the dark in the presence of 100 mM iodoacetamide (IAA) in 10 mM ammonium bicarbonate. After alkylation, the gel pieces were dehydrated twice with 100% ACN for 5 min. To perform in-gel digestion of proteins, 10 µl of trypsin solution (10 ng/ µl trypsin in 50% ACN/10 mM ammonium bicarbonate) was added to the gels, followed by incubation at room temperature for 20 min. To keep the gels immersed throughout digestion, 20 µl of 30% ACN was added and incubated at 37°C for overnight. To extract peptide digestion products, 30 µl of 50% ACN in 0.1% formic acid was added into the gels and incubated at room temperature for 10 min in a shaker. Peptides extracted were collected, dried by vacuum centrifuge and kept at −80°C for further mass spectrometric analysis.

### HCTultra LC-MS/MS analysis

Peptide solutions were analyzed using a HCTultra PTM Discovery System (Bruker Daltonics Ltd., U.K.) coupled to an UltiMate 3000 LC System (Dionex Ltd., U.K.). Peptides were separated on a nanocolumn (PepSwift monolithic column 100 µm i.d.×50 mm). Eluent A was 0.1% formic acid and eluent B was 80% acetonitrile in water containing 0.1% formic acid. Peptide separation was achieved with a linear gradient from 10% to 70% B for 13 min at a flow rate of 300 nl/min, including a regeneration step at 90% B and an equilibration step at 10% B, one run took 20 min. Peptide fragment mass spectra was acquired in data-dependent AutoMS (2) mode with a scan range of 300–1500 *m*/*z*, 3 averages, and up to 5 precursor ions selected from the MS scan 50–3000 *m*/*z*.

### Protein quantitation and identification

For protein quantitation, DeCyder MS Differential Analysis software (DeCyderMS, GE Healthcare) [19] was used. Acquired LC-MS raw data was converted to mzXML format using CompassXport and the PepDetect module was used for automated peptide detection, charge state assignments, and quantification based on the peptide ions signal intensities in MS mode. The analyzed MS/MS data from DeCyderMS was submitted to database searching using the MASCOT software version 2.2 (Matrix Science, London, UK). The data were searched against the NCBI database for protein identification. Search parameters were as follows: Trypsin was selected as the enzyme, with three potential missed cleavage, carbamidomethylated cysteine as fixed modification and oxidation of methionine residues as a variable modification. Peptide mass tolerance was 1.2 Da, fragment mass tolerance was 0.6 Da. ESI ion trap was selected for the instrument type. Identified protein with at least 2 peptides from MASCOT was accepted to be a true match. Over-expressed proteins at least two times across time points and protein ID score > 10 was used as the criterion. The MultiExperiment Viewer (MeV, version 4.6.1) software was used to show maps of protein levels corresponding to the sequences of over-expressed protein profile in *O. viverrini*-infected hamsters in Table 1. The molecular function and biological process was assigned to the protein identified according to the Uniprot database (http://www.uniprot.org).

**Table 1:** Over-expressed proteins in the plasma of *Opisthorchis viverrini*-infected hamsters are listed based on their involvement in biological process and protein score.

Protein name	Biological process	Protein ID Score	Database ID no.	Species	Peptide sequence	Number of peptide
Fibronectin	Structural	46.0	gi|2497975	Pleurodeles waltl	R.TYVITGLQPGTDYK.I	4
MORN repeat-containing protein 4	Structural	23.5	gi|30520314	Homo sapiens	CSAIVQR	2
Developmental pluripotency associated 4	Structural	16.7	gi|40555838	Mus musculus	ILTKSLEG	5
A-kinase anchor protein 11	Structural	15.3	gi|73989321	Canis familiaris	VSPTLPR	2
Nesprin 1	Structural	14.4	gi|23097308	Canis familiaris	VNDLKELTK	15
Cadherin 5	Structural	13.5	gi|18146997	Sus scrofa	GPGLIETASK	2
Kinesin-like protein KIFC2	Structural	12.9	gi|38454244	Rattus norvegicus	DPNGAR	6
Collagen alpha-1(III) chain	Structural	11.7	gi|226423933	Mus musculus	GGPGSPGPK	8
Protocadherin gamma B1	Structural	11.3	gi|126291006	Homo sapiens	SGTVEIR	2
Formin-like protein 1	Structural	11.1	gi|118136288	Mus musculus	KPIQTKFR	16
Kinesin-like protein KIF26B	Structural	10.8	gi|124430752	Homo sapiens	AAQKLNLSSK	6
Protocadherin beta 3	Structural	10.4	gi|17483924	Macaca mulatta	VVAVDG	2
Integrin alpha 7	Structural	10.4	gi|73968265	Canis familiaris	GADVQK	2
MHC class I antigen	Immune response	21.3	gi|32306917	Aotus vociferans	AAAQLGGVNLR	2
Major histocompatibility complex, class I, B	Immune response	21.0	gi|55700782	Macaca mulatta	MQVMAPR	11
H-2 class I histocompatibility antigen	Immune response	17.1	gi|122130	Mus musculus	VMVHDSHSLA	2
Interferon-gamma receptor beta chain	Immune response	17.1	gi|114684351	Pan troglodytes	GGPCAPCAR	2
Interferon alpha 12	Immune response	15.2	gi|28893507	Mus musculus	TLSSSAKLLAR	3
Cathepsin P	Immune response	13.9	gi|28194645	Mesocricetus auratus	MHNGEDAQGR	4
Interferon alpha 8	Immune response	13.3	gi|220442	Mus musculus	ALSSSAKLLAR	4
Immunoglobulin G heavy chain constant region	Immune response	12.1	gi|5902982	Trichosurus vulpecula	CQACDVVGPSVFLFPPNPK	2
Alpha-1-acid glycoprotein 2 (AGP 2)	Immune response	11.3	gi|6754950	Mus musculus	YVGGVK	4
WD repeat domain 78	Cell cycle	26.9	gi|20809864	Mus musculus	STAEAAISKEELEK	3

Protein name	Biological process	Protein ID Score	Database ID no.	Species	Peptide sequence	Number of peptide
N-alpha-acetyltransferase 50	Cell cycle	26.9	gi|21312422	Mus musculus	IEPADAHVLQK	17
PEST proteolytic signal-containing nuclear protein	Cell cycle	23.7	gi|71480098	Mus musculus	KASAISIR	6
Myosin-Id	Cell cycle	19.5	gi|51100974	Homo sapiens	VIVQQPGER	19
Cell cycle regulator Mat89Bb homolog	Cell cycle	14.7	gi|71153011	Mus musculus	METTENGK	13
Caspase 14	Cell cycle	11.9	gi|47169570	Mus musculus	ACLEIR	5
GTP-binding nuclear protein	Cell cycle	10.9	gi|194207255	Equus caballus	SYLMGEFEK	2
Zinc finger protein 415	Transcription	23.1	gi|109125908	Macaca mulatta	ATMAPK	2
PHD finger protein 11	Transcription	19.6	gi|94681065	Mus musculus	VPFLKK	8
Zinc finger protein ZNF223	Transcription	17.0	gi|6118383	Canis familiaris	IQPEMK	8
Polymerase-associated protein RTF1 homolog	Transcription	15.4	gi|20521862	Homo sapiens	HGNDQR	19
Lysine-specific demethylase NO66	Transcription	15.2	gi|7670372	Mus musculus	MDELPNGNGAALLK	2
Snf2-related CBP activator protein	Transcription	11.9	gi|34327954	Homo sapiens	IVCNMRTQFPDLR	13
GON-4-like protein	Transcription	11.5	gi|28972794	Mus musculus	QLPVLVR	10
Protein tyrosine phosphatase	Signal transduction	23.5	gi|187228	Homo sapiens	R.QPPSPK.Q	2
CLC-7 chloride channel protein	Signal transduction	20.4	gi|1177440	Homo sapiens	SQLIVLLK	20
RhoGEF and PH domain containing 6	Signal transduction	16.1	gi|148689626	Mus musculus	GGVAGIQAAK	2
Inversin	Signal transduction	16.1	gi|73949740	Canis familiaris	KPVAHK	5
G protein-coupled receptor 153	Signal transduction	11.8	gi|73956764	Canis familiaris	QYLQVPPTR	2
Collagen/fibrinogen domain-containing protein 1	Signal transduction	10.6	gi|194226003	Equus caballus	QFAKYGSFK	2
Acyl-CoA synthetase family member 4	Metabolism	17.6	gi|30348962	Mus musculus	CVDASPLLVR	3
Glycosyltransferase	Metabolism	17.0	gi|146284146	Mus musculus	R.GEGVDALFVSK.G	22
E3 ubiquitin-protein ligase RNF123	Metabolism	14.3	gi|54027904	Mus musculus	QLMMSLLR	20
Phospholipase A2	Metabolism	12.5	gi|21431612	Mus musculus	XAKFLSYK	2
Deoxyribonuclease-2-beta isoform 1	Metabolism	12.4	gi|194473657	Homo sapiens	SEQLMNDTK	4
Tetratricopeptide repeat domain 39A	Metabolism	10.3	gi|3413866	Homo sapiens	SGTCDILRDR	3
Syntaxin 11	Transport	19.7	gi|71043714	Rattus norvegicus	MSNQAEMK	2
Sodium/glucose cotransporter 5	Transport	17.0	gi|59709496	Sus scrofa	MLPMGLMVMPGMISR	2
Thioredoxin domain-containing 2	Transport	15.2	gi|194214486	Equus caballus	TMQPKESDTLSSSAK	2

Protein name	Biological process	Protein ID Score	Database ID no.	Species	Peptide sequence	Number of peptide
B-spectrin 1	Transport	14.4	gi|40644420	Muscardinus avellanarius	LLGXEDLLQK	3
Sideroflexin 4	Transport	13.4	gi|119569789	Homo sapiens	MKMFPALPR	2
Solute carrier family 23	Transport	11.4	gi|44680145	Homo sapiens	DPSTPLPTEPK	7
60S ribosomal protein L29	Translation	20.5	gi|27671155	Rattus norvegicus	ALVKPQTIK	2
Pumilio 1 isoform 1	Translation	19.5	gi|73950042	Canis familiaris	HVVGVK	3
Ataxin 2	Translation	14.5	gi|1679684	Homo sapiens	RSAAAAPR	9
Matrix metalloproteinase 11	Proteolysis	15.4	gi|78099008	Mus musculus	VDNPVPR	6
Granzyme G precursor	Proteolysis	12.9	gi|53026	Mus musculus	HGTNDIMLLK	3
Carboxylesterase 2F	Biotin biosynthesis	12.5	gi|120474987	Mus musculus	GNHDKIK	3
Fibrinogen gamma chain	Blood coagulation	40.6	gi|222985	Mus musculus	EGFGHLSPTGTTEFWLGNEK	20
DNA/RNA-binding protein KIN17	Stress response	13.1	gi|33859684	Mus musculus	MTDSGDR	3
Microtubule-associated tumor suppressor candidate 2	Tumor suppressor	15.4	gi|71891683	Homo sapiens	ASSSNAAK	7
Unnamed protein product	Unknown function	20.1	gi|26338173	Mus musculus	GKGNCGER	3
Hypothetical protein	Unknown function	14.4	gi|126334366	Monodelphis domestica	DCMKVLR	2
Hypothetical protein	Unknown function	14.0	gi|14017811	Homo sapiens	STWNALSPK	17
Hypothetical protein	Unknown function	11.6	gi|83642812	Mus musculus	EPLCSK	5

Only protein with proteins ID score > 10 and increased expression at least two times across time points are listed.

### Western blot analysis

To validate candidate marker proteins, twenty micrograms of plasma protein was separated on a SDS-PAGE and transferred to a polyvinylidene difluoride membrane (PVDF, Amersham Bioscience, Piscataway, NJ, USA) for 2 h at 60 V. The membrane was blocked with 5% nonfat dried milk powder in phosphate-buffer saline with 0.1%Tween−20 (PBS-T, pH 7.5) for 1 h at room temperature, and then incubated overnight at 4°C with rabbit polyclonal anti-fibronectin or rabbit polyclonal anti-PTPα antibodies diluted in 2% nonfat dried milk-PBS-T (each 1∶3000, ab2413 and ab77792, Abcam, Cambridge, U.K, respectively). After washing with 0.1% PBS-T, the membrane was incubated for 1 h at room temperature with a 1∶3,000 dilution of horseradish peroxidase (HRP)-conjugated secondary antibody (GE Healthcare) diluted in 2% nonfat dried milk/PBS-T. The immunoreactive material was visualized by enhanced chemiluminescence using ECL Western blotting Detection Reagent (GE Healthcare). The intensity of bands of *O. viverrini* infected group was compared to that of un-infected control at the same time points post-treatment. Relative band intensity was compared by using ImageQuant TL software v2005 (1.1.0.1) (non-linear Dynamics, Durham, NC).

### Histopathological study and immunohistochemical staining

The liver tissues were deparaffinized in xylene and then hydrated through a graded series from ethanol to water. For antigen retrieval, sections were floated on 10 mM citric acid buffer (pH 6.0) and then heated at 95–100°C for 10 min and left to stand for 30 min at room temperature. The heat-treated sections were washed twice for 5 min each with PBS (pH 7.4). Before staining, endogenous peroxidase activity was eliminated by incubation for 15 min in 3% hydrogen peroxide in PBS. After washing in PBS, specimens were blocked with 5% fetal bovine serum (Invitrogen, New York, America) for 30 min at room temperature and then incubated overnight at 4°C with 1∶200 rabbit polyclonal anti-fibronectin or 1∶100 rabbit polyclonal anti-PTPα antibodies diluted in PBS (ab2413 and ab77792, Abcam, Cambridge, U.K., respectively). After washing with PBS, the sections were incubated for 1 h at room temperature with horseradish peroxidase-conjugated goat anti-rabbit IgG antibody (1∶200, GE Healthcare). After washing in PBS, stained sections were visualized with 3,3-diaminobenzidine tetrahydrochloride as a chromogen for approximately 5 min. The sections were washed in distilled water and counterstained with hematoxylin for 1 min.

The degree of PTPα and fibronectin expression was evaluated independently by three investigators and were scored as the expression index, in that the percentage of positively stained of inflammatory cells and fibroblasts in the extracellular matrix of periductal fibrosis at 400× magnification (10 randomly selected fields) was calculated and graded according to the scores; grade 0, <30%; grade 1, 30–70% and grade 2, >70% of liver tissues areas [20].

### Statistical analyses

The data were expressed as mean ± S.D. To compare the expression level of protein in infected group compare with normal group, statistical significance of bands intensity was determined using student's *t*-test. A non-parametric Mann-Whitney *U* test was used to compare the grading score of fibronectin and PTPα. Statistical analyses were performed using SPSS version 11.5. *P* values less than 0.05 were considered statistically significant.

## Results

### Plasma proteomics analysis

**Figure 3:**
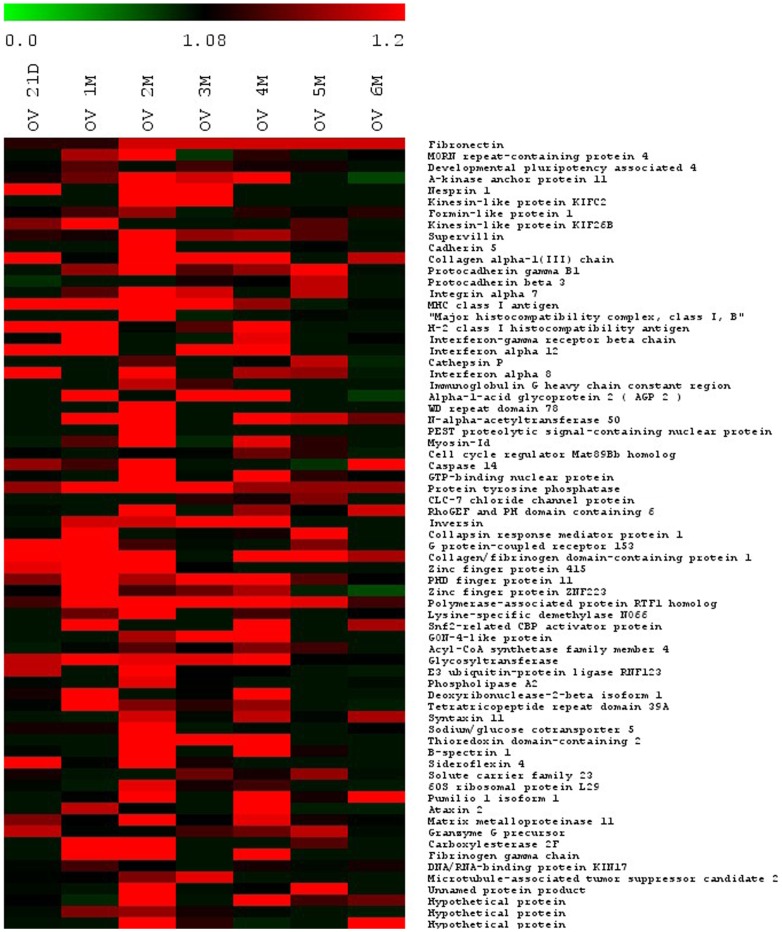
Time-profile expression pattern of 67 differentially up-regulated proteins in the plasma of *Opisthorchis viverrini*-infected hamsters. An over expression pattern of 67 proteins was performed with MultiExperiment Viewer (MeV, version 4.6.1) software. All quantitative information is marked using a color scale ranging from green, dark and red for no changes, a little, and the highest up-regulation ratio, respectively, in expression pattern of OV-infected compare to the normal group at 21 D, 1 M, 2 M, 3 M, 4 M, 5 M, and 6 M post-infection. D, days; M, month(s); OV, *Opisthorchis viverrini.*

Proteins extracted from pooled plasma samples of normal and *O. viverrini*-infected groups were resolved by SDS-PAGE on 7.5% and 12.5% polyacrylamide gels (Figure 2). Each gel slice was independently subjected to in gel tryptic digestion and the resulting peptides were analyzed by LC-MS/MS. A total of 2,957 non-redundant protein identifications were made from 50 µg of protein. To find candidate proteins for biomarker discovery, we focused on over-expressed proteins. Sixty seven proteins were selected for further analysis based on at least two unique tryptic peptides with protein ID score >10 and increased expression at least two times across time points. The alteration of enriched proteins of time in *O. viverrini*-infected hamsters compared to control was shown using MultiExperiment Viewer (MeV, version 4.6.1) software (Figure 3). Details of the 67 proteins and their biological function are presented in Table 1. Highly abundant proteins, with more than 20 peptide matches, included i.e., CLC-7 chloride channel protein, E3 ubiquitin-protein ligase RNF123, glycosyltransferase and fibrinogen gamma chain. These proteins fell into broad functional categories including structural, immune system etc. Among the 67 over-expressed proteins, fibronectin had the highest protein ID score.

### Distribution of biological function of over-expressed proteins

**Figure 4:**
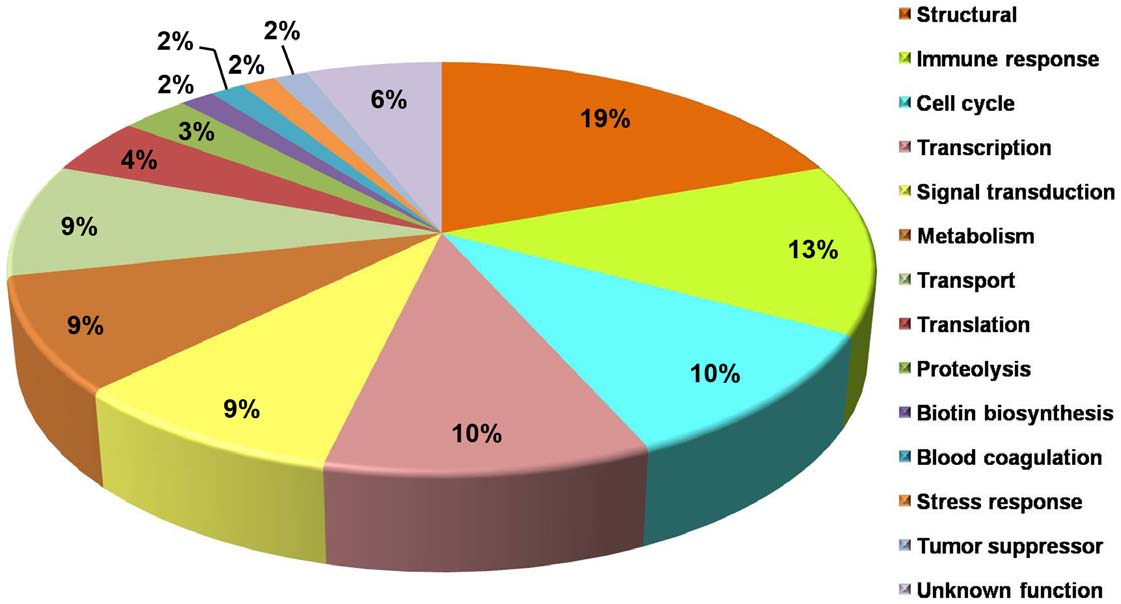
Functional annotation of 67 over-expressed proteins in the plasma of *Opisthorchis viverrini*-infected hamsters classified by biological function. The molecular function and biological process was assigned to the protein identified according to the Uniprot database (http://www.uniprot.org).

The 67 over-expressed proteins were classified according to their biological functions (Figure 4). The majority of assigned functions of over-expressed proteins were structural (19%), immune response (13%), cell cycle (10%), and transcription (10%). Other functions included signal transduction (9%), metabolism (9%), transport (9%), translation (4%), proteolysis (3%), biotin biosynthesis (2%), blood coagulation (2%), stress response (2%), tumor suppressor (2%), and unknown function (6%).

### Validation of protein expression in hamster plasma specimens by western blot analysis

**Figure 5:**
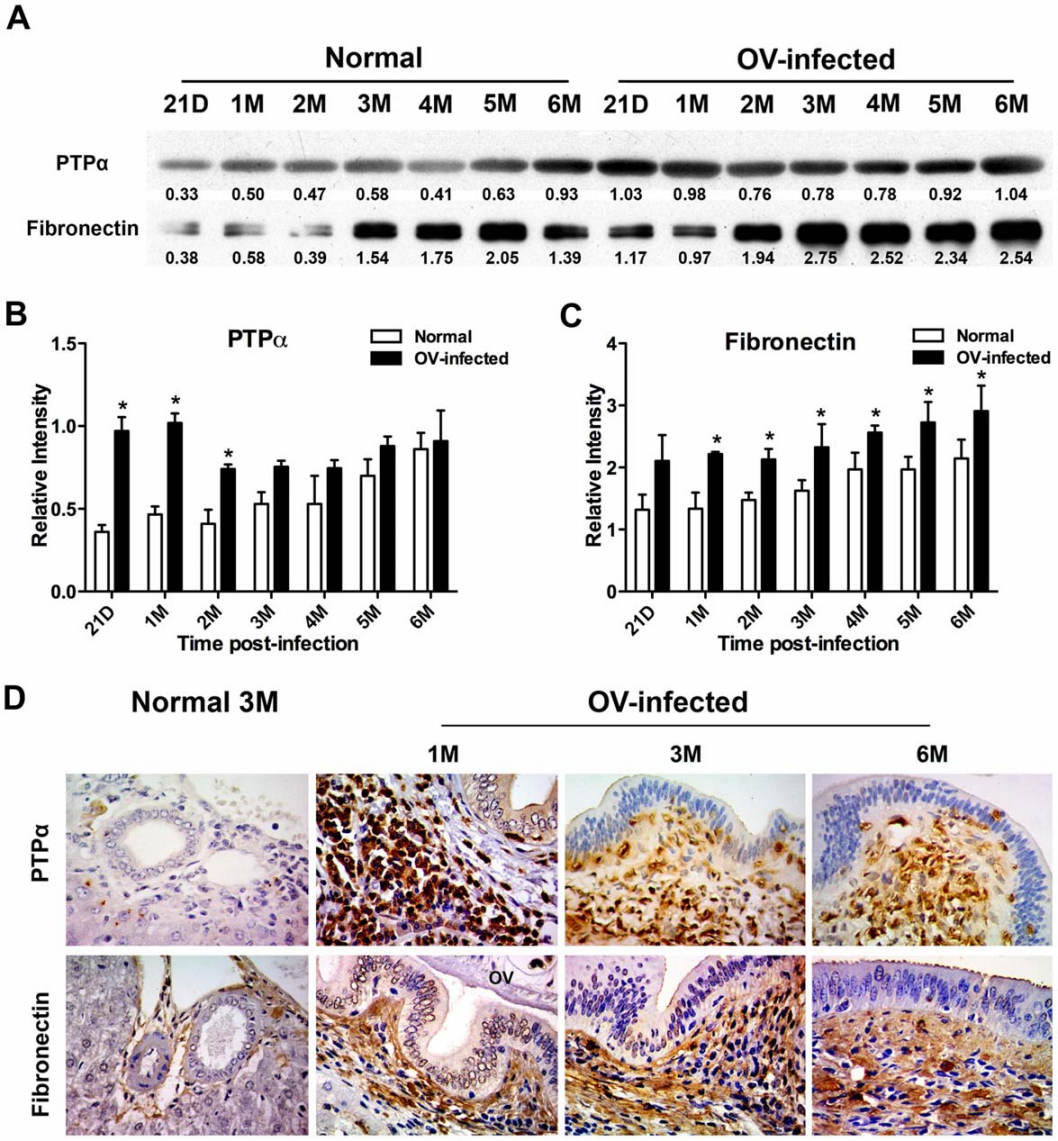
Validation of protein tyrosine phosphatase alpha (PTPα) and fibronectin in the plasma and the liver tissue of *Opisthorchis viverrini*-infected hamsters. Western blot was used to confirm an expression of PTPα and fibronectin (A) protein in the plasma of OV-infected group compared to normal hamsters. Independent experiments were performed for all hamsters (n = 5). Consistent results from at least 3 animals were accepted, and only one gel obtained from one hamster is shown as representative of each group. The number below gel represents relative band intensity, and the relative expression level of PTPα (B) and fibronectin (C) is shown. Immunohistochemical staining shows an expression of PTPα and fibronectin protein in the liver of OV-infected group at 1M, 3M and 6M compared to the normal group at 3M (D). One hamster liver section is representative for five animals in each experimental group. All pictures were taken with same magnification (original 400x). D, days; M, month(s); OV, *Opisthorchis viverrini*.

Based on the assigned functions, protein score and the pattern of overexpression, fibronectin and protein tyrosine phosphatase (PTP) proteins were selected for validation in the plasma of *O. viverrini*-infected hamsters and normal control groups by western blot analysis. Since protein tyrosine phosphatase alpha (PTPα) belongs to human PTP family, and based on the commercial antibody reactivity for both rodent and human proteins, we verified PTPα instead of PTP. PTPα showed a time course of over-expression in plasma that was mirrored in proteomic analysis. As in proteomics analysis, a significant increase was observed at 21 days, expression reached a peak at 1 month and then decreased from 2 months onwards (Figure 5A and 5B). Similarly, expression of fibronectin, assessed using western blotting, matched the proteomic analysis with an over-expression beginning at 1 month and increasing over all time points afterward (Figure 5A and 5C). In addition, the expression of PTPα in uninfected hamsters did not increase over time. No statistically significant increase in fibronectin level was observed at any time point in uninfected control hamsters.

### Validation of protein expression in the hamsters liver tissue specimens by immunohistochemistry

To detect the expression of fibronectin and PTPα proteins in hamster liver, immunohistochemistry was used to validate the distribution expression in tissue sections. These proteins, from the structural and signal transduction groupings, have possible roles in the accumulation of periductal fibrosis. Using specific antibody to fibronectin and PTPα, the expression of PTPα was observed mainly in the cytoplasm of inflammatory cells while fibronectin was observed mainly in the cytoplasm of fibroblasts, the extracellular matrix and some large inflammatory cells (macrophage-like cell) in periductal fibrosis areas. Compared to the normal control, the expression of PTPα significantly increased at 21 days, reached the maximal level at 1 month (grading score 2.00±0.01, *P*<0.05) and then decreased at 3 and 6 months post-infection (each, grading score 1.33±0.58, *P*<0.05). Significantly increased expression of fibronection was observed at 1 month (grading score 0.80±0.01, *P*<0.05) and expression continued to increase over 3 months (grading score 1.40±0.55, *P*<0.05) and 6 months post-infection (grading score 2.00±0.01, *P*<0.01). In addition, slight staining of these markers was found at the portal triad area at the base of the arteriole and bile duct in the normal liver (Figure 5D).

### Characteristics of the human subjects

Plasma samples were collected from healthy volunteers (n = 18), and positive for *O. viverrini*-eggs (n = 39; male  =  21 and female  =  18). The ultrasonography revealed that the grading scores for 0, 1+, 2+ were 20, 16 and 3 patients, respectively.

### Fibronectin and PTPα as the markers of human opisthorchiasis

**Figure 6:**
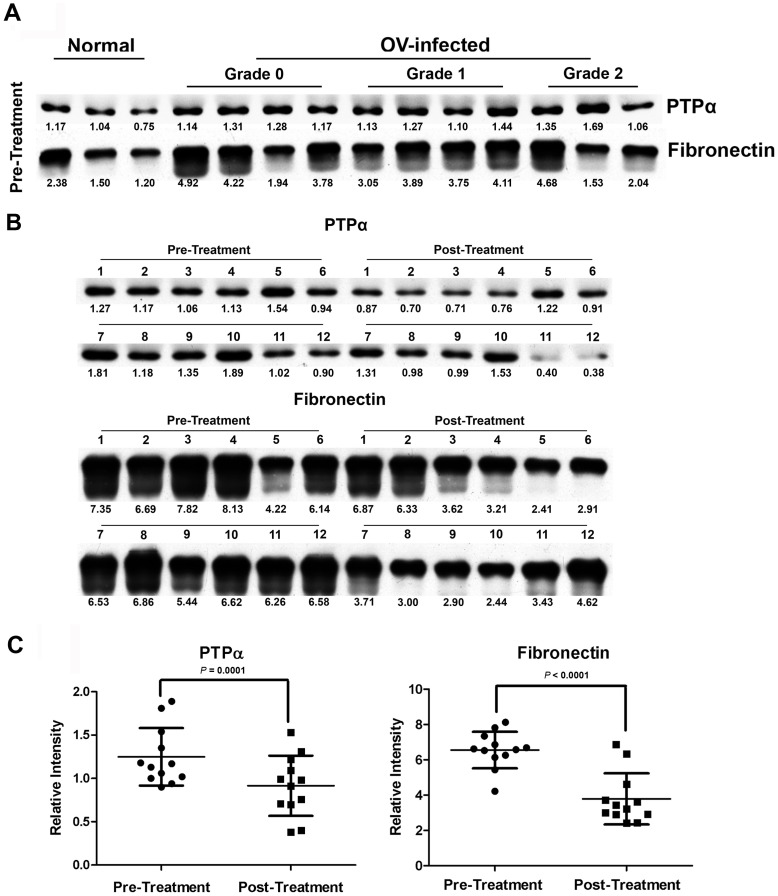
Western blot analysis of protein tyrosine phosphatase alpha (PTPα) and fibronectin in the human plasma samples. Equal protein was subjected to SDS-PAGE followed by immunoblotting using antibody against to PTPα and fibronectin proteins. Densitometry values are shown below of each band. Duplicate independent experiment was performed. (A) Three representative plasma samples of OV-infected patients with difference grading score (grade 0, 1+, and 2+) of periductal fibrosis evaluated by ultrasonography is presented in comparison with normal biliary system. (B) Twelve representative plasma samples of OV-infected patients with grading score 1+ shows between pre- and at 2 months post-treatment. (C) Relative bands intensity of PTPα and fibronectin in 12 OV-infected patients between pre- and post-treatment. The data are shown as mean ± S.D using pair *t*-test. OV, *Opisthorchis viverrini.*

To assess the utility of fibronectin and PTPα as biomarkers in human subjects, western blotting was used to evaluate expression levels of these proteins in plasma from infected humans. Additionally, expression levels were assessed in infected individuals before and 2 months post-treatment with praziquantel. In *O. viverrini* infection, the intensity of fibronectin (average 3.45±1.15 *vs* 1.86±0.51, *P*<0.05) and PTPα (average 1.28±0.18 *vs* 1.05±0.02, *P*<0.05) expression levels were significantly higher than in uninfected individuals. However, expression of these markers was not different among hepatobiliary status (grading scores 0, 1+, and 2+) (Figure 6A). In hepatobiliary grading score 1+, the expression level of PTPα (n = 12, 1.25±0.33 *vs* 0.91±0.34, *P* = 0.0001) and fibronectin (n = 12, 6.56±1.03 *vs* 3.78±1.45, *P*< 0.0001) were significantly decreased by praziquantel treatment (Figure 6B and 6C).

## Discussion

Proteomics analysis is a powerful tool not only for the identification of diagnostic markers of disease, but also for staging of parasitic disease progression [21]. In this study, we have utilized proteomic techniques to search for candidate markers of host protein associated with opisthorchiasis. To do so we first studied the plasma proteome of hamsters infected with *O. viverrini* and consequently validated in the opisthorchiasis patients. Sixty seven proteins were identified as over-expressed at least two times across time points. The major class of over-expressed protein was that possessing a structural function, although inflammatory proteins were also highly represented. Two of the over-expressed proteins, PTPα and fibronectin, were consequently confirmed as over-expressed in both experimental and human opisthorchiasis compared to uninfected controls using western blotting. Moreover, expression of these proteins significantly decreased in infected humans after praziquantel treatment. Therefore, these two proteins may have a biological role in opisthorchiasis and might serve as diagnostic markers of *O. viverrini* infection.

Structural proteins were the most frequently over-expressed proteins in the plasma of *O. viverrini*-infected hamsters. This supports previous observations that, in hamsters, periductal fibrosis increases over time [13], and also increases in hamsters with CCA induced by the combination of *O. viverrini* infection and *N*-nitrosodimethylamine administration [9]. Furthermore, in humans, advanced periductal fibrosis, evaluated by ultrasonography, is currently the most powerful tool for diagnosing opisthorchiasis [7]. On the basis of the protein score fibronectin appeared to one of structural proteins identified, but, other over-expressed structural proteins, involved in the extracellular matrix, were also identified. These included the MORN repeat-containing protein 4, developmental pluripotency associated 4, a-kinase anchor protein 11, nesprin 1, cadherin 5, kinesin-like protein KIFC2, collagen alpha-1(III) chain, protocadherin gamma B1, formin-like protein 1, kinesin-like protein KIF26B, protocadherin beta 3 and integrin alpha 7. These proteins may be associated with opisthorchiasis and, in particular, the structural changes associated with periductal fibrosis. Previously, we have identified actin, a structural protein, in hamsters liver infected with *O. viverrini* [8], but this protein was not detected in this study, possibly due to different source and technique used and actin depolymerization in plasma [22] of opisthorchiasis.

From the structural proteins over-expressed in *O. viverrini* infected hamsters, fibronectin, one of the main components of the extracellular matrix (ECM), was selected for further validation in the plasma and liver. Fibronectin is a large ECM glycoprotein that exists as a dimer and is one of the first ECM proteins to undergo up-regulation during liver fibrosis [23]. Fibronectin is expressed in numerous cell types, including myofibroblast, and can exist in a soluble form present in plasma and various body fluids or as a cellular fibronectin that is confined to tissues [24]. Fibronectin is also expressed by inflammatory cells in response to cytokines and/or growth factors such as TGF-β [24] and, previously, we have demonstrated that expression of TGF-β gene is associated with the accumulation of fibrosis in the hamster model [13]. In the current study, increasing levels of fibronectin were observed over time in the cytoplasm of fibroblasts, the ECM and macrophage-like cells in the areas of periductal fibrosis, and these increases were also detectable in the plasma. Interestingly, fibronectin levels were reduced by praziquantel treatment, suggesting that it may serve as a diagnostic marker for the chronic bile duct disease induced by liver fluke infection, as well as for other chronic hepatic diseases [25].

As a general marker, it has been noted that the circulating levels of cellular fibronectin become elevated during injury or disease [26] and, given the injury and fibrosis caused by adult worms, it is not surprising that a similar mechanism is operating during *O. viverrini* infection. As a marker for parasitic infection, fibronectin was the only early stage marker of granulomatous formation in *Schistosoma haematobium* and *S. mansoni* infection [27] and has been proposed as a potential diagnostic plasma marker in Chagas' disease by using protein array mass spectrometry [28]. Interestingly, fibronectin has been proposed to play a role in the extensive remodeling of the ECM during carcinogenesis [29] and has been found to be either up-regulated [30] or down-regulated [31] in different cancers. Given the progression of opisthorchiasis into CCA, it is of considerable interest to determine whether fibronectin plays a role in cholangiocarcinogenesis and whether this molecule could be utilized as a broad marker to follow the progression of CCA -beginning with infection, through advanced periductal fibrosis and finally carcinogenesis. Given the ubiquity of fibronectin in injury and disease the specificity of a marker based on this protein may not be high, but, in the context of an area of endemic *O. viverrini* infection, it could be useful at initial screening for possible infection and may provide a basis to detect the possibility of the onset of periductal fibrosis and ultimately CCA.

Secreted parasite antigens [32] stimulate the host response by the induction of inflammatory cell migration [5], [33] and inflammatory cell responses to these antigens during inflammation. Alterations in the levels of signal transduction proteins is, therefore, likely to play a major role in host-parasite interaction in *O. viverrini* infection and may be exploited for early biomarker discovery [34], [35] as well as potential therapeutic targets [36], [37]. In our study, a number of protein expression changes associated with the host response to *O. viverrini* were identified in plasma for the first time. In particular changes in the expression levels of effector proteins such as PTPα, CLC-7 chloride channel protein, rhoGEF and PH domain containing 6, inversin, G protein-coupled receptor 153, and collapsin response mediator protein 1 were also identified. These proteins mediate a range of processes - for example, accumulation of CLC-7 chloride channel protein is associated with lysosomal pathology, osteopetrosis [38] and hepatocellular function [39]. CLC-7 is also a potential therapeutic target for the treatment of osteoporosis [40]. In general, signaling protein levels could be expected to increase early as a first response to parasite antigens (Figure 3) and for PTPα, this would appear to be the case. Using western blotting, this protein was first detected at day 21 and reached its peak expression one month post-infection (Figure 5A and 5B). Given the complexity of the host/parasite interactions, the identification of up-regulated proteins involved in gene expression and cellular control mechanisms not only provides a suite of potential early diagnostic markers but may also point the way to novel drug target for control of opisthorchiasis.

Signaling platforms that modulate the inflammatory response are new targets for drug development [41], and the PTP superfamily is a key regulatory component in many inflammatory pathways [42], [43]. In this work, expression of PTPα was observed mainly in the cytoplasm of inflammatory cells of hamster livers infected with *O. viverrini*. The expression level of PTPα increased early in the host-parasite interaction, suggesting it is involved in the acute phase of *O. viverrini* infection. PTPα was also detected at increased levels in the plasma of opisthorchiasis patients and it was significantly decreased by drug treatment, suggesting circulating levels of the protein might be used as a diagnostic marker for opisthorchiasis. In protozoan parasites, host PTPα plays an important role in *Leishmania donovani* [44] and *Trypanosoma brucei* [45] infection, endemic in America and Africa. PTPα induction by *Leishmania* correlates with higher nitric oxide (NO) production [46]. Relevantly, in *O. viverrini*-infected hamsters, we have demonstrated NO-mediated oxidative and nitrative DNA damage in a model of inflammation-mediated carcinogenesis [5]. Overproduction of free radicals including NO and reactive oxygen species (ROS) may therefore participate in the induction of PTPα expression [42] at the early stage of *O. viverrini* infection.

### Conclusion

We have identified several proteins involved in *O. viverrini* infection that might be associated with opisthorchiasis. Among these, fibronectin and PTPα may be useful in the diagnosis of *O. viverrini* infection. In addition, these 2 candidate proteins have been proposed to be useful as drug target treatment in other models. We therefore suggest that PTPα, at an early stage, and fibronectin, at the late stage, may be potential diagnostic markers and for novel drug target discovery of inflammation-mediated diseases including in *O. viverrini* infection in the future. Proteomics based analysis starting from animal to humans may assist in discovering biomarkers [47]. Although the hamster genome has not been fully sequenced and short gun proteomic produced low Mascot Scoring [48], a hamster model of opisthorchiasis may provide a useful strategy to identify candidate markers applicable to human opisthorchiasis with potential utility for a novel diagnostic marker discovery.
